# *Helicobacter pylori* among symptomatic Egyptian children: prevalence, risk factors, and effect on growth

**DOI:** 10.1186/s42506-019-0017-6

**Published:** 2019-05-24

**Authors:** Yasmine Samir Galal, Carolyne Morcos Ghobrial, John Rene Labib, Maha Elsayed Abou-Zekri

**Affiliations:** 1 Departments of Public Health and Community Medicine, Faculty of Medicine, Cairo University, 31 Mohamed Hassan El-Gamal Street, Nasr City, Cairo, 11759 Egypt; 2 Pediatrics, Faculty of Medicine, Cairo University, 115 El Manial Street, Cairo, 11451 Egypt; 3 Pediatrics, Faculty of Medicine, , Cairo University, 7 Riyad Abdel Kader, Nasr City, Cairo, 11759 Egypt; 4 Pediatrics, Faculty of Medicine, , Cairo University, 47 El-Sudan Street, Dokki, Giza, 13211 Egypt

**Keywords:** *Helicobacter pylori*, Prevalence, Growth parameters, Malabsorption, Iron-deficiency anemia

## Abstract

**Aim:**

This study was carried out to determine the prevalence and predictors of *Helicobacter pylori* (*H. pylori*) infection among symptomatic children and the effect on growth.

**Methods:**

A cross-sectional study was conducted in the Outpatient Clinics of the Gastroenterology Unit of the Pediatric Hospital, Cairo University. A total of 630 children complaining of any symptoms or signs suspicious of *H. pylori* infection were enrolled. Weight and height were measured for each child, and the standard deviation scores (*Z*-scores) for weight-for-age (WAZ), weight-for-height (WHZ), and height-for-age (HAZ) were calculated. *H. pylori* was diagnosed using a stool antigen test.

**Results:**

The overall prevalence of infection was 64.6%. Socio-demographic variables significantly associated with *H. pylori* were residence in Upper Egypt (*P* = 0.013) and rural areas (*P* = 0.004), illiteracy of mothers (*P* = 0.017), household crowding index ≥ 3 (*P* = 0.011), absence of pure water supply (*P* = 0.005), and eating from street vendors (*P* < 0.001). Values of WAZ [median, interquartile range (IQR) in infected = − 1.3(− 2.7 to − 0.4) and non-infected = − 0.7(− 2.1 to − 0.1), *P* < 0.001], HAZ (*P* = 0.036), and WHZ (*P* = 0.005) were significantly lower in children infected with *H. pylori*. After performing a backward logistic regression analysis, eating from street vendors (OR = 1.879, 95% CI 1.346–2.625, *P* < 0.001), absence of pure water supply (OR = 1.725, 95% CI 1.162–2.561, *P* = 0.007), and overcrowding (OR = 1.547, 95% CI 1.100–2.177, *P* = 0.012) remained the significant predictors of *H. pylori* infection.

**Conclusion:**

A high prevalence of *H. pylori* infection among symptomatic children was detected. The extra-digestive effects of *H. pylori* were revealed in the form of affection of growth parameters and reduced levels of serum hemoglobin, iron, and ferritin.

## Introduction

*Helicobacter pylori* (*H. pylori*) is one of the most common chronic infections with a worldwide prevalence of about 50% [1]. This infection is predominantly acquired during early childhood especially in poor countries [2, 3]. The prevalence of *H. pylori* varies markedly between countries [4]; about 50% of children are infected by 10 years of age in developing countries [5].

The mode of transmission of *H. pylori* through the fecal-oral or oral-oral routes [6] was supported by the evidence that most risk factors for infection are closely related to poor living conditions including lower socio-economic status, bad hygiene, deficiency of sanitation, household crowding, bed sharing, and food- and water-borne transmission [7–9].

*H. pylori* has been recognized as a causative agent of chronic gastritis [10, 11]. However, extra-digestive effects on growth parameters have been reported in young children. A wide range of studies has stated an association between *H. pylori* infection and childhood growth impairment [12–14]. One mechanism is that *H. pylori* causes depressed gastric acid secretion, which could result in infection with enteropathogens leading to diarrhea, malabsorption of nutrients, reduced food intake as a result of dyspepsia, and iron-deficiency anemia (IDA) [15, 16]. However, proving the role of *H. pylori* alone in childhood growth impairment is difficult where numerous confounding variables such as socio-economic status and diet also exist [17].

Despite that *H. pylori* infection constitutes a public health problem often with serious complications especially in a developing country like Egypt, few studies were conducted revealing high rates of infection in both adults [18, 19] and the pediatric age groups [20, 21].

Since evidence from the literature postulates that *H. pylori* infection occurs mostly during childhood [2], studying the epidemiology of this infection in pediatric patients can enable better understanding of the risk factors and consequences of infection. Therefore, this study was conducted in the Pediatric Hospital of Cairo University to determine the prevalence of *H. pylori* infection among children, to identify the different predictors that may influence the acquisition of infection and the effect on growth.

## Material and methods

### Study design, period, and setting

This was a cross-sectional, hospital-based study conducted in the Outpatient Clinics of the Gastroenterology Unit of the Pediatric Hospital, Cairo University. The study took place over a 6-month period from February till July 2017. About 500–600 patients per month attend the outpatient clinic with different gastrointestinal problems.

### Study population

All patients attending the Outpatient Clinics of the Gastroenterology Unit during the study period and complaining of any symptoms or signs suspicious for *H. pylori* infection (abdominal pain, anemia not responding to treatment, recurrent vomiting, and upper gastrointestinal bleeding) were recruited. A total of 630 children ≤ 18 years were enrolled for the study. Children with history of previous use of antibiotics or proton-pump inhibitors at least 2 weeks before attending the outpatient clinics were excluded.

### Study tools and data collection

#### Demographic details and clinical features

- A pre-test structured questionnaire was used to collect information from the children’s parents regarding possible risk factors for infection including the following: (i) socio-demographic characteristics including age, gender, residence, mother education (non-educated were illiterates and educated were those who completed any stage of education), household crowding index (number of people living in the household/number of rooms), and availability of potable water; and (ii) dietary practices, such as daily eating from street vendors.
  Household crowding index: the house was considered crowded when the number of persons living in the house divided by the number of rooms was ≥ 3.
  Questions used in this section were adopted from the available literature [6, 20] by the researchers and were revised by three experts from Public Health and Pediatrics to check content validity. Pilot testing: the preliminary data collection form was tested on 20 cases to assess the clarity and comprehension of questions and the time needed to answer the questionnaire.
- Patients’ records were reviewed for the following: (i) presenting symptoms and signs (abdominal pain, vomiting, hematemesis, melena, and pallor) and (ii) history of medications—proton pump inhibitors and antibiotics.

### Anthropometric measurements

Weights were measured using electronic scales, and heights were measured using length boards. For children over 2 years, height sticks were used. Then, the standard deviation scores (*Z*-scores) for weight-for-age (WAZ), weight-for-height (WHZ), and height-for-age (HAZ) were calculated using the Center of Disease Control (CDC) online calculator program. [22]

### Laboratory tests

Blood samples were collected from each child and sent to the Clinical and Chemical Pathology Department at the Faculty of Medicine, Cairo University, to measure serum hemoglobin, serum iron and ferritin, liver function tests [alanine transaminase (ALT), aspartate transaminase (AST), total bilirubin, and albumin], and coagulation profile [prothrombin time (PT), prothrombin concentration (PC), and international normalized ratio (INR)].

### Helicobacter pylori testing

*H. pylori* was diagnosed using a stool antigen test which is rapid, non-invasive, reliable, easy to perform and can be used to detect an existing infection. Participants were given clean, leak proof containers to provide fresh stools within 2 h; the children and/or their parents were asked to identify the time and date of specimen collection. All stool specimens were collected and transported in a cold (− 4 °C) specimen carrier to the laboratory. Specimens that were expected to stay more than 2 h before processing were frozen at (− 20 °C). A lateral flow immunochromatographic assay for detection of *H. pylori* antigen in stool was used with a sensitivity of 96% and specificity of 83%. [23] Based on the intensity of the color developed, results were reported as *H. pylori* antigen not detected, equivocal, or detected [Onsite *H. pylori* Rapid Ag test].

### Statistical analysis

Pre-coded data was entered on the computer using the Statistical Package of Social Science Software Program (SPSS), version 23, to be statistically analyzed. Data was presented using mean, median, range, and interquartile range for quantitative variables and frequency and percentages for qualitative ones. Comparison between groups was performed using Mann Whitney test for quantitative variables and chi-square test for qualitative ones. Backward stepwise logistic regression model was conducted to explore the predictors of *H. pylori* infection. *P* values less than 0.05 were considered statistically significant.

## Results

Table 1 shows the socio-demographic data, dietary practices, and predictive clinical features among the studied population. A total of 630 children, 325 males (51.6%) and 305 females (48.4%), were enrolled for the study. The total number of *H. pylori*-positive individuals was 407 (64.6%). The participants’ age ranged from 1 to 15 years with a median of 7 years. The highest prevalence of *H. pylori* infection was among children > 10 years (32.9%), while the lowest was among those < 3 years (13.8%), with no statistical differences. Apart from age and sex, the following socio-demographic variables were significantly less associated with *H. pylori* infection: residence in urban areas (OR = 0.615, 95% CI 0.442–0.854, *P* = 0.004), literacy of mothers (OR = 0.669, 95% CI 0.481–0.930, *P* = 0.017), crowding index ≥ 3 (OR = 1.545, 95% CI 1.105–2.160, *P* = 0.011), and presence of pure water supply (OR = 0.577, 95% CI 0.391–0.852, *P* = 0.005). Eating from street vendors was significantly associated with positive *H. pylori* infection (OR = 1.914, 95% CI 1.375–2.663, *P* < 0.001).

**Table 1:** Association of *H. pylori* infection among symptomatic Egyptian children with sociodemographic, personal, and clinical data

	Stool antigen for *H. pylori*		Total	*p* value^#^	OR	95% CI
	+VE (*n* = 407)	−VE (*n* = 223)				
Age groups						
< 3 years	56 (13.8%)	33 (14.8%)	89	Ref	Ref	Ref
3–6 years	102 (25.1%)	72 (32.3%)	174	0.500	0.835	0.494–1.412
7–10 years	115 (28.3%)	43 (19.3%)	158	0.107	1.576	0.905–2.745
> 10 years	134 (32.9%)	75 (33.6%)	209	0.845	1.053	0.629–1.762
Sex						
Male	205 (50.4%)	120 (53.8%)	325	0.408	0.871	0.628–1.208
Female	202 (49.6%)	103 (46.2%)	305	Ref	Ref	Ref
Region						
Upper Egypt	186 (45.7%)	79 (35.4%)	265	0.013	1.534	1.096–2.148
Lower Egypt	221 (54.3%)	144 (64.6%)	365	Ref	Ref	Ref
Residence						
Urban	161 (39.6%)	115 (51.6%)	276	0.004	0.615	0.442–0.854
Rural	246 (60.4%)	108 (48.4%)	354	Ref	Ref	Ref
Mother education						
Educated	157 (38.6%)	108 (48.4%)	265	0.017	0.669	0.481–0.930
Not educated	250 (61.4%)	115 (51.6%)	365	Ref	Ref	Ref
Crowding index						
≥ 3	270 (66.3%)	125 (56.1%)	395	0.011	1.545	1.105–2.160
< 3	137 (33.7%)	98 (43.9%)	235	Ref	Ref	Ref
Pure water supply	283 (69.5%)	178 (79.8%)	461	0.005	0.577	0.391–0.852
Eating from street vendors	246 (60.4%)	99 (44.4%)	345	< 0.001	1.914	1.375–2.663
Symptoms and signs						
Abdominal pain	310 (76.2%)	142 (63.7%)	452	0.001	1.823	1.277–2.602
Vomiting	24 (5.9%)	0 (0%)	24	< 0.001	NA	NA
Hematemesis	378 (92.9%)	177 (79.4%)	555	< 0.001	3.387	2.059–5.573
Melena	182 (44.7%)	99 (44.4%)	281	0.938	1.013	0.729–1.407
Pallor	39 (9.6%)	7 (3.1%)	46	0.003	3.270	1.438–7.439

*+VE* positive, *−VE* negative, *Ref* reference, *OR* odds ratio, *CI* confidence interval, *NA* not applicable

^*#*^Chi square test

Clinical symptoms and signs significantly associated with positive *H. pylori* infection included abdominal pain (OR = 1.823, 95% CI 1.277–2.602, *P* = 0.001), vomiting (*P* < 0.001), hematemesis (OR = 3.387, 95% CI 2.059–5.573, *P* < 0.001), and pallor (OR = 3.270, 95% CI 1.438–7.439, *P* = 0.003) (Table 1).

The values of WAZ [median (IQR) in infected = − 1.3(− 2.7 to − 0.4) and non-infected = − 0.7(− 2.1 to − 0.1), *P* < 0.001], HAZ [median (IQR) in infected = − 1.3(− 2.2 to 0.1) and non-infected = − 0.9(− 2.0 to 0.1), *P* = 0.036], and WHZ [median (IQR) in infected = − 1.2(− 2.9–0.3) and non-infected = − 0.3(− 2.1–0.5), *P* = 0.005] were significantly lower in children (whole sample) infected with *H. pylori* infection compared with children free of infection (Table 2).

**Table 2:** Comparison between *H. pylori*-infected and non-infected symptomatic children regarding anthropometric measurements and laboratory investigations

	Stool antigen for *H. p*ylori among males				
	+VE (*n* = 205)		−VE (*n* = 120)		*p* value^@^
	Median	IQR	Median	IQR	
Males WAZ	− 1.1	− 2.5 to − 0.4	− 1.0	− 2.2 to − 0.3	0.052
HAZ	− 1.6	− 2.3 to − 0.7	− 1.4	− 2.2 to 0.0	0.219
WHZ	− 1.2	−2.3 to 0.5	−0.2	− 1.7 to 0.5	0.103
Hemoglobin [gm/dl]	10.5	9.4–11.4	11.0	9.8–13.0	< 0.001
Ferritin [ng/dl]	15.1	9.0–29.5	41.0	16.0–60.0	< 0.001
Serum iron [ug/dl]	42.0	27.0–70.0	41.0	32.0–54.0	0.832
INR	1.1	1.0–1.2	1.0	1.0–1.1	0.152
ALT	33.0	28.0–59.0	33.0	26.5–49.0	0.935
AST	45.0	41.0–68.0	44.0	39.0–82.0	0.862
Serum albumin	3.8	3.4–4.3	3.9	3.7–4.4	0.043
Females WAZ	− 1.4	− 2.8 to − 0.2	− 0.5	− 2.1 to 0.1	0.003
HAZ	− 0.9	− 2.0 to 0.4	− 0.4	− 1.2 to 0.2	0.097
WHZ	− 1.5	− 3.6 to − 0.5	− 0.3	− 2.6 to 0.2	0.006
Hemoglobin [gm/dl]	10.6	9.5–11.1	11.2	10.5–13.0	< 0.001
Ferritin [ng/dl]	25.3	8.6–60.0	38.0	15.1–78.0	0.027
Serum iron [ug/dl]	37.9	25.0–74.0	46.0	39.0–80.0	0.002
INR	1.0	1.0–1.1	1.1	1.0–1.2	0.010
ALT	31.0	27.0–49.0	28.0	25.0–49.0	0.045
AST	45.0	35.0–73.0	38.0	32.0–82.0	0.033
Serum albumin	4.1	3.6–4.3	3.9	3.6–4.1	0.096
The whole sample WAZ	− 1.3	− 2.7 to − 0.4	− 0.7	− 2.1 to − 0.1	< 0.001
HAZ	− 1.3	− 2.2 to 0.1	− 0.9	− 2.0 to 0.1	0.036
WHZ	− 1.2	− 2.9 to 0.3	− 0.3	− 2.1 to 0.5	0.005
Hemoglobin [gm/dl]	10.6	9.4–11.2	11.0	10.1–13.0	< 0.001
Ferritin [ng/dl]	17.5	9.0–60.0	41.0	15.1–60.0	< 0.001
Serum iron [ug/dl]	41.0	25.0–71.0	46.0	34.0–70.0	0.015
INR	1.0	1.0–1.1	1.0	1.0–1.2	0.453
ALT	31.0	27.0–59.0	31.0	25.0–49.0	0.191
AST	45.0	36.0–68.0	44.0	34.0–82.0	0.242
Serum albumin	3.9	3.5–4.3	3.9	3.6–4.4	0.585

*IQR* interquartile range, *WAZ* weight-for-age *Z* score, *HAZ* height-for-age *Z* score, *WHZ* weight-for-height *Z* score, *INR* international normalized ratio, *ALT* alanine transaminase, *AST* aspartate Aminotransferase

^@^Mann Whitney test

**Fig. 1:**
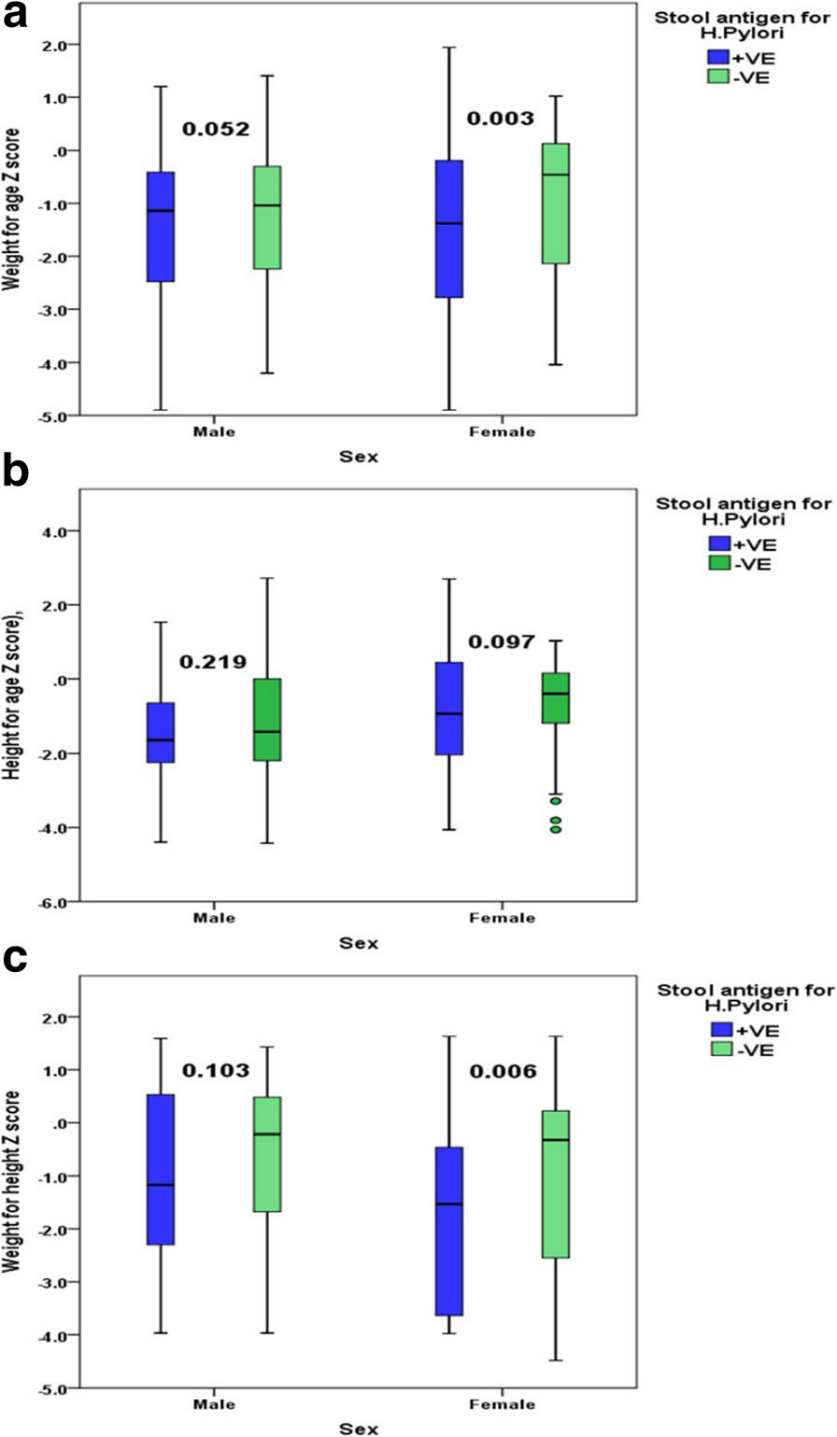
**a** Box plot showing distribution of WAZ in relation to sex and *H. pylori* infection. **b** Box plot showing distribution of HAZ in relation to sex and *H. pylori* infection. **c** Box plot showing distribution of WHZ in relation to sex and *H. pylori* infection

Figure 1 a, b, c illustrates the anthropometric measurements (WAZ, HAZ, and WHZ) in relation to infection status and sex. These values did not show significant decline within *H. pylori*-infected males when compared to non-infected ones. However, *H. pylori*-infected females showed significant decline in both WAZ (median (IQR): infected = − 1.4 (− 2.8 to − 0.2) and non-infected = − 0.5 (− 2.1 to 0.1); *P* = 0.003) and WHZ (median (IQR): infected = − 1.5 (− 3.6 to − 0.5) and non-infected = − 0.3 (− 2.6 to 0.2); *P* = 0.006) but not in HAZ (median (IQR): infected = − 0.9 (− 2.0 to 0.4) and non-infected = − 0.4 (− 1.2–0.2); *P* = 0.097).

Regarding laboratory investigations, levels of serum hemoglobin (median, interquartile range (IQR): infected = 10.6 (9.4–11.2) and non-infected = 11.0 (10.1–13.0); *P* < 0.001), ferritin (median (IQR): infected = 17.5 (9.0–60.0) and non-infected = 41.0 (15.1–60.0); *P* < 0.001), and iron (median (IQR): infected = 41.0 (25.0–71.0) and non-infected = 46.0 (34.0–70.0); *P* = 0.015) were significantly reduced in infected children. No significant association was found between infected and non-infected children as regards INR, liver enzymes, and serum albumin (Table 2).

Backward stepwise logistic regression model was conducted to explore predictors of positive *H. pylori* infection (Table 3). In the last step, only eating from street vendors (OR = 1.879, 95% CI 1.346–2.625; *P* < 0.001), absence of pure water supply (OR = 1.725, 95% CI 1.162–2.561; *P* = 0.007), and overcrowding (OR = 1.547, 95% CI 1.100–2.177; *P* = 0.012) remained the significant predictors of *H. pylori* infection.

**Table 3:** Backward stepwise logistic regression model for predictors of *H. pylori* infection among symptomatic Egyptian children

	*β*	*p* value	Adjusted OR	95% CI
Eating from street vendors	0.631	< 0.001	1.879	1.346–2.625
Absence of pure water supply	0.545	0.007	1.725	1.162–2.561
Overcrowding	0.436	0.012	1.547	1.100–2.177
Constant	− 0.134	0.424	0.875	–

*β* beta coefficient, *OR* odds ratio, *CI* confidence interval)

## Discussion

To the authors’ best knowledge, this is the first study conducted in the Pediatric Hospital at Cairo University to detect the prevalence and predictors of *H. pylori* infection among children, and the effect of infection on children’s growth parameters. The current study showed a high prevalence (64.6%) of *H. pylori* infection among symptomatic children attending the Outpatient Clinics of the Gastroenterology unit. This result is lower than that observed in previous studies among children in Egypt, which reported *H. pylori* infection in 72.4% and 68% of children [21, 24]. However, it is higher than that detected in other countries of the Eastern Mediterranean region like Saudi Arabia, where a 49.8% prevalence of *H. pylori* infection was detected among symptomatic children [25]. This variation of infection rates between studies is probably due to differences in the study design, patient inclusion criteria, sample size, and techniques used for *H. pylori* detection.

In this study, the prevalence of *H. pylori* was found to increase with age; children > 10 years had the highest *H. pylori* infection rates (32.9%), while those < 3 years had the lowest (13.8%). Similarly in a study conducted by Hasosah et al. [25], the prevalence of *H. pylori* was 10.5% among children < 3 years old; however, it reached 57.7% among those > 10 years old. This could be explained by increased children’s contact with the community and outdoor exposure with practicing faulty dietary practices like eating from street vendors. In contrast, other studies found that infection increases in early childhood due to bed or bedroom sharing with an infected sibling [7, 26]. In accordance with other studies [25, 27], gender was not found to be a risk factor for childhood infection with *H. pylori* in the present study.

Children from rural areas had a significantly higher infection rates than those from urban areas (60.4% vs. 39.6%, *P* < 0.004). Consistent with the findings of the present study, Sayed et al. reported a higher seroprevalence of *H. pylori* infection in rural areas in Assiut Governorate than in urban areas [18]. This could be attributed to the poor socio-economic status, poor sanitation, bad hygienic behaviors, and absence of sources of pure water supply among people living in rural areas.

Educational level has been known as an important determinant of *H. pylori* prevalence in both developed and developing countries [28]. In the current study, illiteracy of mothers was significantly associated with positive *H. pylori* infection; this could be explained by the assumption that the mothers’ educational status reflects on the healthy habits and hygienic behavior acquired by their children. This goes in accordance with a study conducted by Malaty et al., where children whose mothers did not complete high school had higher rates of *H. pylori* infection compared to those whose mothers had a partial or complete college education [29]. However, Awuku et al. found no significant association between the educational level of either parents and the prevalence of *H. pylori* infection [6].

Overcrowded conditions especially in developing countries have been reported as a major risk factor for *H. pylori* infection, because that creates closer contact between mothers and children and between siblings that might spread the infection to each other [30]. In this study, household crowding index ≥ 3 was significantly associated with positive *H. pylori* infection (*P* = 0.011).

Another important environmental risk factor that has been linked to *H. pylori* spread in previous studies is the absence of a source of pure water supply [6, 31]. Similarly, our study found that a significantly higher percent of non-infected children had a source of pure water supply compared to infected children (79.8% vs. 69.5%, *P* = 0.005). Moreover, the role of food-borne transmission of *H. pylori* has been stated in the literature [32], especially in developing countries where bad sanitary conditions exist such as the presence of flies, bad hygiene, and eating fruits and vegetables without washing them or eating uncooked vegetables. In agreement with that, the present study detected a significant association between eating from street vendors where sanitary measures are not guaranteed and positive *H. pylori* infection (*P* < 0.001).

In this study, abdominal pain, vomiting, hematemesis, and pallor were among the significant clinical predictors of *H. pylori* infection. Similarly, in a study conducted in Saudi Arabia, children with abdominal pain were 2.39 times more likely to have *H. pylori* infection [25].

There is noticeable controversy in the findings of studies examining the effect of *H. pylori* infection on growth in children, as some illustrated the presence [13, 33] of association and others denied it [34, 35]. However, new evidence has emerged recently from the studies of Mera et al. [36] and Yang et al. [37] who reported a beneficial effect of *H. pylori* eradication on the growth of children. In the current study, the values of WAZ, WHZ, and HAZ were significantly reduced in infected children compared to non-infected ones. However, distribution of these measurements according to infection status and sex revealed significant reduction in WAZ and WHZ values in infected females compared to non-infected ones; non-affection of the HAZ could be explained by early detection of *H. pylori* infection before affecting the chronic malnutrition index (HAZ). However, there were no significant differences in values of WAZ, WHZ, and HAZ between infected and non-infected males, which may be explained by gender discrimination and deeply rooted traditions and beliefs where more attention is given to male infants especially in a developing country like Egypt. Similarly, in a study conducted in Egypt to examine the effect of *H. pylori* infection on children growth [21], the prevalence of stunted growth was higher in infected children than in children free of infection; also the values of WAZ and weight-for-age percentile score (WAP) signified reduced growth in children with *H. pylori* compared with children free of infection; however, in that study, the differences in height and weight associated with *H. pylori* infection were more noticeable in boys than girls.

Regarding the association between *H. pylori* infection and other extra-digestive parameters in the current study, significant reduction was observed in the levels of serum hemoglobin, iron, and ferritin among infected children compared to non-infected ones. Similarly, several studies conducted in both developed and developing countries revealed lower serum ferritin and hemoglobin concentrations [38], and/or higher proportion of iron deficiency (ID) or IDA [39, 40] in *H. pylori* positive than in *H. pylori-*negative children. In contrast, other studies failed to prove such association [41].

In this study, variables that were significantly associated with *H. pylori* infection and were retained in logistic regression model and thus could be considered as potential predictors for *H. pylori* infection included eating from street vendors, absence of pure water supply, and overcrowding. In another study conducted by Hasosah et al. [25], variables that remained significant for *H. pylori* infection in the multivariate model were age > 10 years, bed sharing, contact with the community, a high number of family members, and low income.

### Limitations of the study

Among the limitations of this study was the involvement of symptomatic children admitted to the Outpatient Clinics of the Gastroenterology Unit at the Pediatric Hospital, Cairo University, for diagnosis and treatment and not the general population. Also, the socioeconomic and behavioral risk factors were reported by the children’s parents and thus may not be so accurate.

## Conclusion

This study revealed a high prevalence of *H. pylori* infection (64.6%) among symptomatic children attending the Outpatient Clinics of the Pediatric Hospital, Cairo University. Residence in rural areas, educational status of mothers (non-educated), household crowding index ≥ 3, absence of pure water supply, and eating from street vendors were significantly associated with *H. pylori* infection. After conducting a stepwise logistic regression analysis, only eating from street vendors, absence of pure water supply, and overcrowding remained the significant predictors of infection. Also, the extra-digestive effects of *H. pylori* were demonstrated; the values of growth parameters (WAZ, HAZ, and WHZ) and levels of serum hemoglobin, iron, and ferritin were significantly lower in infected children. Based on the findings of this study, it is recommended that diagnostic and treatment programs for *H. pylori* be considered a public health priority especially in a developing country like Egypt. Further studies among asymptomatic children are also needed to tackle this problem.

## Abbreviations

ALT: Alanine transaminase
AST: Aspartate transaminase
CDC: Centers of Disease Control and Prevention
CI: Confidence interval
*H. pylori*: *Helicobacter pylori*
HAZ: Height-for-age
ID: Iron deficiency
IDA: Iron-deficiency anemia
INR: International normalized ratio
IQR: Interquartile range
NA: Not applicable
OR: Odds ratio
PC: Prothrombin concentration
PT: Prothrombin time
SPSS: Statistical Package of Social Science
WAP: Weight-for-age percentile
WAZ: Weight-for-age
WHZ: Weight-for-height
*β*: Beta coefficient
